# Explaining variation in the kinship composition of mammal groups

**DOI:** 10.1093/beheco/arae032

**Published:** 2024-04-17

**Authors:** Mark Dyble

**Affiliations:** Department of Archaeology, University of Cambridge, Downing Street, CB2 3DZ Cambridge, United Kingdom

**Keywords:** cooperation, kinship, mammal societies, pair-bonding, reproductive skew, siblings

## Abstract

Variation in cooperative behavior across mammals is strongly related to the kinship composition of groups. Although the factors affecting average genetic relatedness within groups have been studied, the factors that contribute to the production of different categories of kin remain underexplored. Here, I use a mathematical model to explore the factors that determine the proportion of full siblings, maternal half-siblings, paternal half-siblings, and non-siblings within mammal groups. The results suggest that the production of paternal half-siblings is increased by high male reproductive skew and a female-biased sex ratio, the production of maternal half-siblings is increased by high female reproductive skew and male-biased sex ratio, and that there are two routes to the production of full siblings: either high reproductive skew in both sexes (as seen in cooperatively breeding species) or pair-bond stability within groups of low reproductive skew (as seen in humans). These results broadly correspond to observed variation in sibling composition across mammals.

## Introduction

Empirical studies have shown that average coefficients of relatedness (*r*) between group members vary widely between animal species and, in mammals, range from close to zero to values exceeding *r* = 0.5, the expected level of relatedness between full siblings (Briga et al. 2012; Dyble and Clutton-Brock 2020; Clutton-Brock 2021; Pereira et al. 2023). Comparative analyses have revealed that these interspecific contrasts in kinship are consistently associated with interspecific differences in social behavior (Griffin and West 2003; Lukas and Clutton-Brock 2018; West et al. 2021) and that, as would be predicted by inclusive fitness theory (Hamilton 1964; Gardner et al. 2011), species exhibiting the most highly developed forms of alloparental care tend to live in social groups within which the average degree of genetic relatedness between groupmates is high (Briga et al. 2012; Lukas and Clutton-Brock 2018). In many species, genetic relatedness also predicts patterns of cooperation within groups, with individuals recognizing and preferentially cooperating with close kin in many species (e.g., Widdig et al. 2001; Green et al. 2015; De Moor et al. 2020).

Given the importance of kinship in determining social behavior, it is important to establish the factors that contribute to variation in kinship structure across mammals. Following more general population structure models (e.g., Johnstone 2008; Lehmann and Rousset 2010), mammal-specific models have identified the key factors influencing variation in mean within-group relatedness, suggesting average kinship between group members is largely a consequence of variation in group size, male and female reproductive skew, litter size, and rates of dispersal (Lukas et al. 2005; Dyble and Clutton-Brock 2020, 2023). However, as argued in a recent paper by Pereira et al. (2023), the focus on mean within-group relatedness in these studies overlooks potentially important variance in the relatedness of individuals to their group mates as well as differences in the proportion of different categories of relatives that occur within groups (the kinship composition).

Understanding the factors that influence the kinship composition of groups (rather than just the average within-group relatedness) is interesting for several reasons. First, kin-discriminate altruism is most likely to evolve in species that live in groups containing a mixture of kin and non-kin (Cornwallis et al. 2009), with low variance in relatedness potentially selecting for indiscriminate cooperative behavior (Duncan et al. 2019). Second, the relative proportions of maternal siblings and paternal siblings are of consequence to the evolution of social behavior since, in many species, maternal siblings are more readily identified than paternal siblings (e.g., Widdig et al. 2001; Langergraber et al. 2007; Widdig 2007, 2013; Perry et al. 2008). Third, transitions to cooperative breeding in animals are more likely to occur in species where full siblings are commonly produced; full siblings are related to one another by *r *= 0.5 on average rather than *r *= 0.25 such that investment in them returns—all else being equal—twice the inclusive fitness benefit (Hughes et al. 2008; Cornwallis et al. 2010; Downing et al. 2020). Finally, understanding the factors that determine the proportion of sibling types is important for understanding human social evolution in particular. From a broader mammalian perspective, humans are unusual in being highly cooperative despite living in groups of low average relatedness (Gintis et al. 2003; Hill et al. 2011; Dyble et al. 2015). One hypothesis is that although within-group relatedness is low, the pair-bond stability seen in humans leads to the production of full siblings and the evolution of highly cooperative family units forming the core of human social organization (Chapais 2008, 2013; Layton et al. 2012). Modeling the effect of pair-bond stability on the kinship composition of groups can allow us to explore this hypothesis more thoroughly.

Here, I expand an existing mathematical model (Dyble and Clutton-Brock 2020) to explore the effect of social and life-history factors on the frequency of full siblings, maternal half-siblings, paternal half-siblings, and non-siblings born into a group, as well as the proportion of offspring sharing at least one grandparent. In doing so, I follow the work of Altmann (1979), who considered the degree of relatedness between maternal siblings and members of a juvenile cohort, showing that the production of maternal half-siblings rather than full siblings is promoted by small litter size, polygynous mating, and turnover in reproducing males and that the production of paternal half-siblings is promoted by the monopolization of reproduction by a single male. Altmann’s (1979) model, however, does not explore the consequences of the production of large litters, unequal reproductive success between breeding males, pair-bonding, or skew in female reproduction, nor does it estimate relatedness between members of different juvenile cohorts. Incorporation of these additional features into a theoretical model is necessary for a more comprehensive understanding of the relative production of different categories of siblings across the full range of variation seen in mammalian life history, mating patterns, and social structure.

Adding kinship composition to the existing model also allows exploration of the relationship between the kinship composition of groups and mean within-group relatedness and to determine what, if anything, can be inferred about the kinship composition of a group from a single point estimate of mean relatedness as reported in many empirical studies. This issue was raised by Pereira et al. (2023), who suggested that mean genetic relatedness is not sufficient to determine whether groups are composed solely of kin or whether they include a mix of kin and non-kin among natal individuals.

## Methods

To explore the effect of various social and life-history factors on the relative frequency of siblings in mammal groups, I extend an existing mathematical model that predicts mean within-group relatedness (Dyble and Clutton-Brock 2020). This model makes a number of simplifying assumptions about demography and dispersal but nonetheless is able to accurately predict variation in mean within-group relatedness across mammals (Dyble and Clutton-Brock 2020). The original model has eleven parameters: the number of adult males (*N*_*m*_), number of adult females (*N_f_*), number of juvenile cohorts in the group at the same time (*n*), male reproductive skew (*α*) probability of subordinate female reproduction (*β*), litter size (*κ*), the probability of dominant male retaining dominance between reproductive cohorts (*τ_m_*), the probability of dominant female retaining dominance between reproductive cohorts (*τ*_*f*_), the number of juveniles per adult (*θ*), male dispersal, and female dispersal. Here, I am concerned with kinship among offspring born into a group and, as such, the number of juveniles per adult (*θ*), male dispersal, and female dispersal are disregarded even though these factors will play a role in determining kinship composition among adults (e.g., female dispersal and male philopatry will be expected to result in higher relatedness among adult male groupmates than among adult female groupmates (Rousset 2004; Johnstone and Cant 2008; Dyble and Clutton-Brock 2020)). Since my intention is to consider variation in kinship composition across mammals in general rather than one mammalian taxa in particular, I consider broad parameter ranges (Table 1) and include, for example, values for the parameters determining female skew (*β* and *τ*_*f*_) that incorporate both the high female skew seen among cooperative breeders and the low female skew seen among plural breeding species including most primates (Hager and Jones 2009; Ross et al. 2023).

**Table 1.** Social and life history parameters explored.

Parameter	Symbol	Range of values explored
Number of adult males	*N*_*m*_	{*N*_*m*_ ∈ *Z* | 2 ≤ *N*_*m*_ ≤ 10}
Number of adult females	*N*_*f*_	{*N*_*f*_ ∈ *Z* | 2 ≤ *N*_*f*_ ≤ 10}
Number of juvenile cohorts	*n*	{*n* ∈ *Z* | 1 ≤ *n* ≤ 6}
Male reproductive skew	*α*	{*α* ∈ *R* | 0 ≤ *α* < 1}
Probability of a subordinate female reproducing	*β*	{*β* ∈ *R* | 0 < *β* ≤ 1}
Litter size	*κ*	{*κ* ∈ *Z* | 1 ≤ *κ* ≤ 6}
Probability of dominant male retaining dominance	*τ*_*m*_	{*τ*_*m*_ ∈ *R* | 0 ≤ *τ*_*m*_ ≤ 1}
Probability of dominant female retaining dominance	*τ*_*f*_	{*τ*_*f*_ ∈ *R* | 0 ≤ *τ*_*f*_ ≤ 1}
Pair-bond stability	*ε*	{*ε* ∈ *R* | 0.5 ≤ *ε* < 1}

Although the original model (Dyble and Clutton-Brock 2020) was produced to predict mean genetic relatedness across a whole group, including adults and juveniles, the core feature of the model is a set of equations that estimate the independent probabilities of two juveniles born into the same group sharing a mother and sharing a father. In the previous work, these probabilities were referred to as *M* and *P*, respectively. Here, I refer to them as *P*(*Mother*) and *P*(*Father*).

### Probability of sharing a father

In the model, the probability of two juveniles sharing a father *P*(*Father*) is determined by the number of breeding males (*N*_*m*_), male reproductive skew (*α*), the number of juvenile cohorts in the group (*n*), and the probability of the dominant male retaining dominance (*τ*_*m*_). I assume that one male is reproductively dominant, taking a proportion of the reproductive success of others, determined by α such that the total proportion of offspring produced by the dominant in any given juvenile cohort (*d*) is *d* = 1/ *N*_*m*_ + *α*(*N*_*m*_ − 1)/ *N*_*m*_. When *α* = 0, all males have an equal probability of fathering an offspring. When *α* = 1, the dominant male sires all offspring born in that cohort. Given *d*, the probability of two individuals born in the same cohort sharing a father (*p*_*0*_) is *d*^2^ + (1 − *d*)^2^/(*N*_*m*_ − 1). When the dominant male retains reproductive dominance between the production of juvenile cohorts (with probability *τ*_*m*_), then *P*(*Father*) is the same as it would be if they were in the same cohort; *p*_*0*_. If there is a turnover in male dominance (with probability 1- *τ*_*m*_) then it is assumed that all males have an equal chance of becoming dominant such that the expected probability of juveniles in successive cohorts sharing a father is 1/ *N*_*m*_. Reproductive skew can take many forms (Johnstone 2000; Hager and Jones 2009; Ross et al. 2023); the model used for male reproductive skew here is chosen primarily for mathematical convenience, although it is close to the ‘priority-of-access’ form where a dominant male has a disproportionate amount of reproductive success and all other males share the remaining reproductive success relatively equally (Altmann 1962). Mathematically, α is not a measure used in empirical studies of reproductive skew, although *α*^2^ is equal to Bradbury’s bounded skew index (Bradbury et al. 1985). Note that although α determines the amount of reproductive skew that occurs among *N*_*m*_ males in the production of a single cohort of offspring, lifetime reproductive skew would also be influenced by the number of males in the group (*N*_*m*_), the number of juvenile cohorts (*n*), and the probability of a dominant male retaining dominance (*τ*_*m*_). Where there is a high degree of turnover in male reproductive dominance, skew in lifetime reproductive success can be relatively low, even if the distribution of paternity within a single juvenile cohort is highly skewed (Lukas and Clutton-Brock 2014; Dyble and Clutton-Brock 2023).

### Probability of sharing a mother

What is the probability of two juveniles sharing a mother, *P*(*Mother*)? This depends on the number of females (*N*_*f*_), litter size (*κ*), the number of juvenile cohorts (*n*), and the probability of subordinate female reproduction (*β*). The probability of two individuals born in the same cohort sharing a mother is:


$$\begin{array}{l} \underset{i=1}{\overset{N_{f}}{\sum }}\;\left(\begin{array}{l} N_{f}-1\;i-1\; \end{array}\right)\beta^{\left(i-1\right)}{\left(1-\beta \right)}^{\left(N_{f}-i\right)}\frac{\kappa -1}{i\kappa -1} \end{array}$$ (1)

The rationale for (1) is as follows. I assume that there will always be a dominant female who breeds and that the other *N*_*f*_—1 females in the group are ‘subordinate’ and breed with probability *β*. When *β* = 1, all females breed and the distinction between dominant and subordinate becomes arbitrary. The probability that two individuals born in the same cohort share a mother will depend on how many females breed. So long as *β *> 0 there is a possibility that all *N*_*f*_ females in the group breed. For two individuals within the same cohort to share a mother, they need to be littermates. For each possible number of breeding females (minimum 1 and maximum *N*_*f*_), the formula considers the expected number of littermates an individual will have (*κ* – 1) and divides this by the total number of other offspring that will be born into the cohort (*iκ* – 1). This gives the probability of two individuals born in the same cohort sharing a mother given a certain number of breeding females, *i*. The formula then weights each of these probabilities by the probability of this number of breeding females occurring which is: $\left(\begin{array}{l} N_{f}-1\;i-1\; \end{array}\right)\beta^{\left(i-1\right)}{\left(1-\beta \right)}^{\left(N_{f}-i\right)}$. As an example, if *κ* = 2, β* *= 0.5, and *N*_*f*_ = 3 then there is 25% probability that only the dominant female will breed and produce a single litter within which the probability of offspring sharing a mother is 100%, a 50% probability that both the dominant and one subordinate female will breed, producing a cohort within which the probability of offspring sharing a mother is 1/3, and a 25% probability that the dominant and both subordinate females will breed, producing a cohort within which the probability of offspring sharing a mother is 1/5. Weighting by the probability of each occurrence, the probability of two individuals born in the same cohort sharing a mother in this scenario is, therefore, 0.47.

If no dominance change occurs between cohorts (with probability *τ*_*f*_), then the probability of two juveniles born in separate cohorts sharing a mother is only slightly different from in the above:


$$\begin{array}{l} \underset{i=1}{\overset{N_{f}}{\sum }}\;\left(\begin{array}{l} N_{f}-1\;i-1\; \end{array}\right)\beta^{\left(i-1\right)}{\left(1-\beta \right)}^{\left(N_{f}-i\right)}\frac{1}{i} \end{array}$$ (2)

The difference between (2) and (1) is that the expected number of maternal siblings in future cohorts is not influenced by litter size, only by the number of breeding females, and thus (*κ* - 1)/(*iκ* – 1) becomes 1/*i.* For simplicity, (2) assumes that the reproducing subordinates are the same in successive cohorts.

If female dominance does change between groups (with probability 1- *τ*_*f*_), then it is assumed that all females have an equal chance of becoming dominant such that the expected probability of a female being the mother of a given offspring after a dominance turnover is 1/ *N*_*f*_. The model for calculating the probability of sharing a mother differs from the above model for the probability of sharing a father in order to incorporate the litter size parameter and to recognize the constraints on maximum female reproductive success in any one reproductive cohort: in this model, females either breed and produce a litter of *κ* offspring, or else do not breed. Low levels of skew under this model (high *β*) produce an egalitarian distribution of female reproductive success. High levels of female skew under this model (low *β*) produce patterns similar to that seen in cooperative breeding species, where a dominant female monopolizes reproduction with only occasional loss of control (or concession) to subordinate females (Clutton-Brock 1998; Duncan et al. 2023). Note that the model considers discrete reproductive cohorts of the kind that exist among seasonal breeders or among non-seasonal breeders in which females synchronize reproduction. Where females breed continuously and asynchronously, discrete reproductive cohorts are not produced, and in these cases, the application of discrete reproductive cohorts represents a simplifying assumption.

### Estimating the proportion of full siblings, maternal half-siblings, and paternal half-siblings

If the probability of two juveniles sharing a father is independent of their probability of sharing a mother, then the probability of them being full siblings would simply be *P*(*Mother*)·*P*(*Father*). Similarly, the probability of them being maternal half-siblings would be *P*(*Mother*)·(1-*P*(*Father*)), and the probability of them being paternal half-siblings would be (1-*P*(*Mother*))·*P*(*Father*). In many species, however, these probabilities are not independent. For example, in litter-bearing mammals, littermates are more likely to share a father than non-littermates, even though multiple-paternity litters do occur in many species (Carpenter et al. 2004; Randall et al. 2007; Dobson et al. 2018). Similarly, in species with female reproductive exclusivity, all offspring within a litter will share the same father, and in species with strong pair-bonds, females typically reproduce with the same male in successive breeding seasons, increasing the proportion of full sibling dyads produced across juvenile cohorts. In these cases, sharing a mother increases the chances that two juveniles share a father. This non-independence is incorporated by adding a new term (*ε*) to the model, which varies 0.5 ≤ *ε* < 1 and increases the extent to which the probability of two offspring sharing a father given that they share a mother, *P*(*Father*|*Mother*), exceeds the baseline probability of them sharing a father, *P*(*Father*). This is achieved by taking the baseline odds of sharing a father *O*(*Father*), which are *P*(*Father*)/(1-*P*(*Father*)), multiplying it by the odds of *ε,* O(*ε),* which are ε/(1- ε), and then converting this to a probability:


$$\begin{array}{l} P\left(Father\;|\;Mother\right)=\frac{O\left(Father\right)*O\left(\varepsilon \right)}{1+\left(O\left(Father\right)*O\left(\varepsilon \right)\right)} \end{array}$$ (3)

When *ε* = 0.5, two juveniles are no more likely to share a father if they share a mother, and when *ε* = 1, all offspring born to the same mother share a father and are full siblings. This term, *ε*, is described as *pair-bond stability*, with pair-bonding used without assuming that reproductive bonds are necessarily monogamous: a male polygynously mating with two females in two successive breeding seasons is thought of here as having a stable pair-bond with each female.

Given the above, the probability of two juveniles being full siblings is *P*(*Father*|*Mother*)·*P*(*Mother*), the probability of two juveniles being maternal half-siblings is *P*(¬*Father*|*Mother*)·*P*(*Mother*), and the probability of two juveniles being paternal half-siblings is *P*(*Father*|¬*Mother*)·*P*(¬*Mother*). In all cases, the required conditional probabilities can be inferred from *P*(*Father*), *P*(*Mother*), and *P*(*Father*|*Mother*) using Bayes’ theorem. *P*(¬*Mother*) and *P*(¬*Father*) denote the probabilities of *not* sharing a mother or father, respectively.

To estimate the proportion of full siblings, half-siblings, and non-siblings among a group of juveniles, we need to consider *P*(*Father*), *P*(*Mother*), and *P*(*Father*|*Mother*) for a pair of juveniles across 5 scenarios: (1) juveniles born in the same cohort, (2) juveniles born in different cohorts between which no change in male or female dominance has occurred, (3) juveniles born in different cohorts with a change in female dominance having occurred between cohorts, (4) juveniles born in different cohorts with a change in male dominance having occurred between cohorts, (5) juveniles born in different cohorts with changes in both female and male dominance having occurred between cohorts. The relevant probabilities for each scenario are listed in Table 2. In scenarios (4) and (5), it is assumed that a male dominance change is disruptive enough to nullify any pair-bond stability such that following a dominance change, it is assumed that *P*(*Father*|*Mother*) = *P*(*Father*). By estimating the relative occurrence of each of the 5 scenarios given the *τ*_*m*_ (probability of dominant male retaining dominance), *τ*_*f*,_ (probability of dominant female retaining dominance), and *n* (number of juvenile cohorts) parameters, I estimate the proportion of full siblings (*P*_*full*_), maternal half-siblings (*P*_*mat*_), paternal half-siblings (*P*_*pat*_), and non-siblings (*P*_*non*_) across a group of juveniles. I assume that the modeled proportions of kin are preserved as the juveniles age such that these proportions are preserved among natal (non-dispersing) adult members of the group. Using these proportions, it is possible to estimate the proportion of juveniles who share at least one grandparent (*P*_*kin*_) as 1-(*P*_*non*_^*2*^). This allows us to follow the definition of kin from Pereira et al. (2023) as those who share at least one parent or grandparent. These individuals would be genetically related by at least *r *= 0.0625.

**Table 2.** Estimating the proportions of siblings across a set of juvenile cohorts. Here, *i* is the difference in the cohort number (e.g., where individuals are from cohorts 1 and 2, *i* = 1, and where individuals are from cohorts 2 and 5, *i* = 3).

Scenario	*P*(*Father*)	*P*(*Mother*)	*P*(*Father*|*Mother*)	Relative frequency of occurrence
1. Within the same cohort	*d * ^2^ + (1 − *d* )^2^/(*N*_*m*_ − 1)	As in Eq1	As in Equation 3	1/*n*
2. Between cohorts, no dominance change	*d* ^ 2^ + (1 − *d* )^2^/(*N*_*m*_ − 1)	As in Eq2	As in Equation 3	$\underset{i=1}{\overset{n-1}{\sum }}\;2\left(n-i\right){\tau_{m}}^{i}{\tau_{f}}^{i}$
3. Between cohorts, female dominance change	*d * ^2^ + (1 − *d* )^2^/(*N*_*m*_ − 1)	1/*N*_*f*_	As in Equation 3	$\underset{i=1}{\overset{n-1}{\sum }}\;2\left(n-i\right){\tau_{m}}^{i}\left(1-{\tau_{f}}^{i}\right)$
4. Between cohorts, male dominance change	1/*N*_*m*_	As in Eq 2	*P*(Father)	$\underset{i=1}{\overset{n-1}{\sum }}\;2\left(n-i\right)(1-{\tau_{m}}^{i}){\tau_{f}}^{i}$
5. Between cohorts, male and female dominance changes	1/ *N*_*m*_	1/ *N*_*f*_	*P*(Father)	$\underset{i=1}{\overset{n-1}{\sum }}\;2\left(n-i\right)(1-{\tau_{m}}^{i})\left(1-{\tau_{f}}^{i}\right)$

### Exploring the effects of social and life-history parameters on kinship

Although this model is deterministic, it is sufficiently complex as to make it difficult to know *a priori* what effect each parameter will have on the outcome variables. In order to examine the effects of the model parameters on the predicted frequency of sibling relationships, I calculated the outcome variables across 10^5^ iterations of the model in which I randomly set parameters within ranges listed in Table 1, with the exception of pair-bond stability (*ε*), as explained below. In so far as I am looking for a set of parameters that produce particular outcomes of interest (such as high frequencies of different categories of siblings), I follow Gallagher et al. (2015) in referring to these as “Fitting to Idealised Outcomes” (FIO) simulations. The results of these FIO simulations allow for the estimation of a general correlation of the effect of each parameter on the outcome measures across a set of all-else-random parameters, as well as the exploration of interactions between parameters. In line with previous studies employing this modeling approach (e.g., Gallagher et al. 2015; Dyble 2021), I estimate these correlations with Spearman’s rank correlation coefficients, rather than Pearson’s correlation coefficients because while relationships between model parameters and outcomes are often monotonic, they are rarely linear. Plotting histograms of the frequency with which different parameter values produce particular outcomes (e.g., the production of a high proportion of full siblings) allows for the identification of any non-linear relationships (Supplementary Fig. S2).

Although the addition of pair-bonding to the model is of theoretical interest, it does produce a complication for the FIO simulations because when the degree of pair-bond stability in the model is high, there are some combinations of random parameter values that result in impossible conditional probabilities (e.g., that *P*(*Father*|*Mother*).*P*(*Mother*) > *P*(*Father*)). To avoid this problem, in the main set of FIO simulations, the pair-bond stability parameter is set to *ε *= 0.5 (no pair-bond stability). I then run a second set of FIO simulations in which pair-bond stability is varied across the range 0.5 ≤ *ε* < 1, and where the range of values for *N*_*f*_ and *β* is restricted to reduce the frequency of impossible combinations (5 ≤ *N*_*f*_ ≤ 10; 0.5 ≤ *β* ≤ 1), with the few remaining impossible parameter combinations excluded (~2% of the total). Relatedness among juveniles is estimated including shared ancestry through grandparents following Dyble and Clutton-Brock (2020) and as set out in the SM.

## Results

### Proportion of siblings within groups

Across simulations, mean relatedness among juveniles ranged from *r* = 0.04 to 0.50, and the proportion of juveniles that are siblings ranged from 10% to 100%. I first explored the relationship between each of the variables and the estimated proportion of juveniles born into groups that are either full or half-siblings. Across all simulations, the proportion of juveniles that are siblings was greater when there were fewer breeding males and females (*N*_*m*_; *r*_*s*_ = −0.31; *N*_*f;*_*r*_*s*_ = −0.38), a higher male reproductive skew (*α*; *r*_*s*_ = 0.50) and a lower probability of subordinate females reproducing (*β; r*_*s*_ = −0.35). The proportion of siblings was more modestly correlated with male reproductive tenure (*τ*_*m*_; *r*_*s*_ = 0.16), female reproductive tenure (*τ*_*f*_ ; *r*_*s*_ = 0.14), the number of juvenile cohorts (*n*, *r*_*s*_ = −0.20), and litter size (*κ; r*_*s*_ = 0.12) (Table 1). These findings correspond to the results of a previous analysis of the relationship between these parameters and mean within-group relatedness (Dyble and Clutton-Brock 2020), and, as would be expected, there is a strong correlation between the proportion of juveniles that are siblings and estimated mean within-group relatedness (*r*_*s*_ = 0.99). The production of social groups in which almost all natal individuals are kin (where > 95% dyads are kin; where kin are defined as individuals who share at least one grandparent, following a recent analysis (Pereira et al. 2023)) is closely associated with mean relatedness among natal individuals: the predictions of the model are that almost all groups with a mean relatedness among natal individuals of *r* < 0.25 would be ‘mix-related’ and almost groups with a mean relatedness of *r* > 0.35 would be “related” (Supplementary Fig. S1). These results broadly conform to the empirical data comparing the classification of species as “related” or “mix-related” with published estimates of mean relatedness for these species (Supplementary Fig. S1).

### Proportion of sibling types

**Fig. 1.**
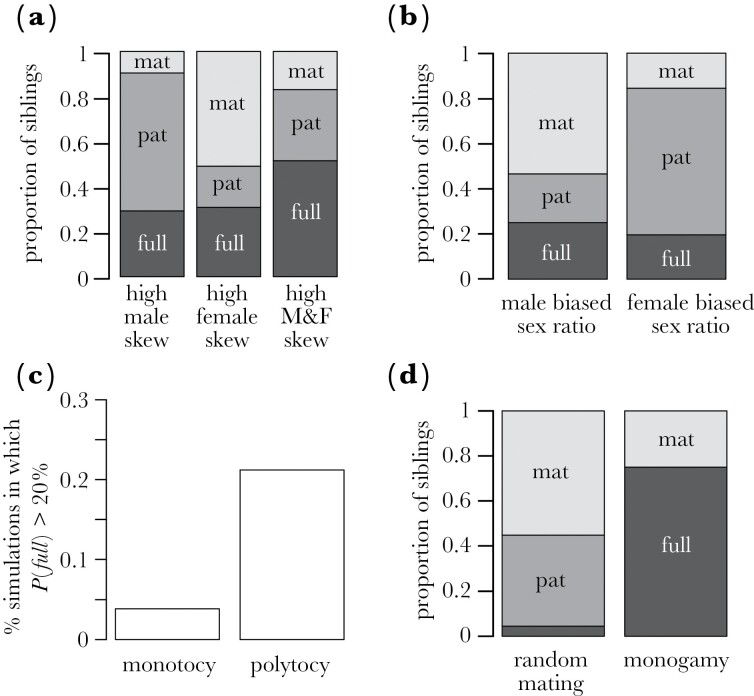
Factors determining the kinship composition of mammal groups. (a) the relative proportion of full siblings (“full,” dark gray), paternal half-siblings (“pat,” gray), and maternal half-siblings (“mat,” light gray) produced under conditions of high male reproductive skew (*α* > 0.75, *τ*_*m*_ > 0.75), high female reproductive skew (*β* < 0.25, *τ*_*f*_ > 0.75), and high reproductive skew among both sexes (*α* > 0.75, *β* < 0.25, *τ*_*m*_ > 0.75, *τ*_*f*_ > 0.75); (b) the relative proportion of siblings produced under conditions of a male biased sex ratio (*N*_*m*_/(*N*_*m*_ + *N*_*f*_) > 0.7) and female biased sex ratio (*N*_*m*_/(*N*_*m*_ + *N*_*f*_) < 0.3); (c) the proportion of simulations in which at least 20% of juveniles were full siblings given monotocy (*κ* = 1) and polytocy (*κ* >=2); (d) illustrative example of the effect of pair-bonding stability: the proportion of siblings produced when *N*_*m*_ = 10, *N*_*f*_ = 10, *n* = 4, α = 0, β = 1, *κ* = 1, *τ*_*m*_ = 1 and under random mating (*ε* = 0.5, left) and pair-bond stability (*ε* = 1, right). Factors determining the kinship composition of mammal groups

**Fig. 2.**
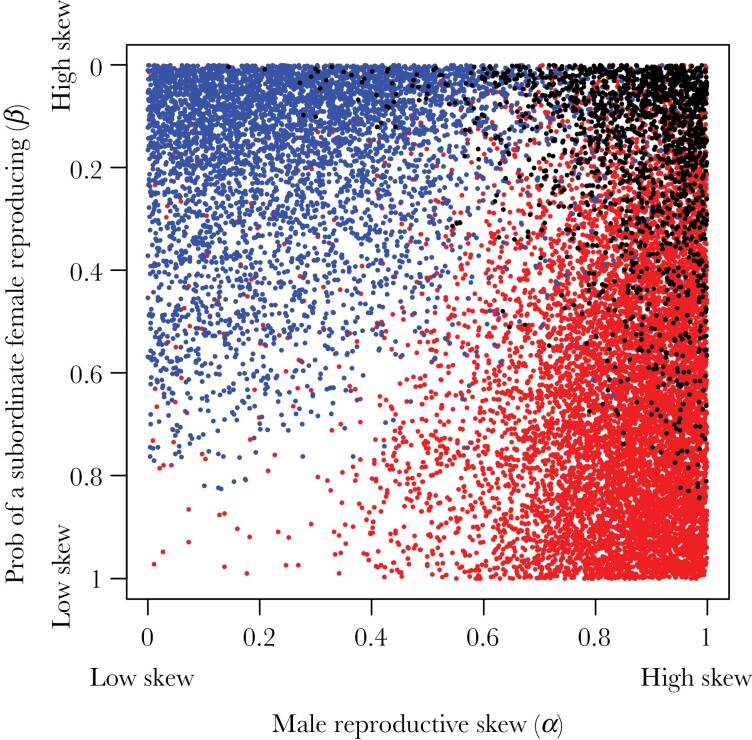
Production of sibling types as a consequence of the interaction between male and female reproductive skew. Dots show values for male reproductive skew (α) and probability of a subordinate female reproducing (β) within the FIO simulations that resulted in > 50% of offspring being paternal half-siblings (red), maternal half-siblings (blue), and full siblings (black). Production of sibling types as a consequence of the interaction between male and female reproductive skew.

Across simulations, reproductive skew is strongly associated with the proportions of sibling types. Specifically, greater male skew and an increased probability of a dominant male retaining dominance increase the production of paternal half-siblings (*α; r*_*s*_ = 0.59; *τ*_*m*_; *r*_*s*_ = 0.16), while greater female skew and an increased probability of a dominant female retaining dominance increase the relative production of maternal half-siblings (*β; r*_*s*_ = −0.46; *τ*_*f*_; *r*_*s*_ = 0.16) (Fig. 1a). Greater reproductive skew and increased probabilities of a dominant individuals retaining their dominance in both males and females are associated with a greater proportion of full siblings (*α; r*_*s*_ = 0.43; *τ*_*m*_; *r*_*s*_ = 0.12; *β*; *r*_*s*_ = −0.43; *τ*_*f*_; *r*_*s*_ = 0.11; Fig. 1a). The interaction between male and female reproductive skew is shown in Fig. 2: when male reproductive skew is high (high *α*), and female reproductive skew is low (high *β*), a large number of paternal half-siblings are produced (red dots), when the female reproductive skew is high (low *β*), and male reproductive skew is low (low *α*), a large number of maternal half-siblings are produced (blue dots), and when the reproductive skew is high in both males and females, a large proportion of full siblings are produced (black dots). Related to this, the relative proportion of paternal versus maternal half-siblings is strongly influenced by sex ratio: a male-biased adult sex ratio reduces the proportion of paternal half-siblings and increases the proportion of maternal half-siblings, while a female-biased sex ratio reduces the proportion of maternal half-siblings and increases the proportion of paternal half-siblings (Fig. 1b). Litter size (*κ*) is positively correlated with the proportion of full siblings (*r*_*s*_ = 0.24) and maternal half-siblings (*r*_*s*_ = 0.18) and negatively correlated with paternal half-siblings (*r*_*s*_ = −0.12) (Table 3). Groups containing a substantial proportion of full siblings are much more likely to occur given polytocy than monotocy. For example, the probability of over 20% of juveniles being full siblings was ~5 times higher given polytocy than under monotocy (3.9% versus 21.2%) (Fig. 1c). The number of juvenile cohorts was positively associated with the proportion of maternal half-siblings (*r*_*s*_ = 0.11) but negatively correlated with the proportion of full siblings (*r*_*s*_ = −0.08) and paternal half-siblings (*r*_*s*_ = −0.11). It is worth noting that the proportion of paternal half-siblings generally exceeded the proportion of maternal half-siblings across simulations; more paternal than maternal half-siblings were estimated in ~60% of parameter combinations. Although this is a consequence of the fact that male reproductive skew more readily exceeds female reproductive skew in the model, this is also true empirically across mammal societies, with levels of male reproductive skew usually exceeding levels of female skew in mammals (Clutton-Brock 1998; Ross et al. 2023). This sex difference in reproductive skew is largely a consequence of the fact that males have a higher potential reproductive rate than females (Clutton-Brock and Parker 1992).

**Table 3.** Spearman’s rank correlation coefficients (ρ) between parameters and measures of relatedness and kinship composition. *As explained above, the correlations for the pair-bond stability parameter were modeled separately. Full results for this second set of simulations are given in Supplementary Table S1 and do not differ qualitatively from the results below. Histograms showing the frequency at which large proportions of each sibling type are produced across the range for each parameter are shown in Supplementary Fig. S2: all of these relationships are monotonic.

Parameter:	Spearman’s rank correlation coefficients with:					
	Mean relatedness	SD in relatedness	% full sibs	% paternal sibs	% maternal sibs	% sibs
Number of males (*N*_*m*_)	−0.31	−0.26	−0.30	−0.40	0.18	−0.31
Number of adult females (*N*_*f*_)	−0.41	−0.37	−0.45	0.29	−0.53	−0.38
Juvenile cohorts (*n*)	−0.18	0.11	−0.08	−0.11	0.11	−0.20
Male reproductive skew (α)	0.50	0.24	0.43	0.59	−0.35	0.50
Female subordinate reproduction (β)	−0.38	−0.31	−0.43	0.32	−0.46	−0.35
Litter size (*κ*)	0.15	0.23	0.24	−0.12	0.18	0.12
Stability of male tenure (*τ*_*m*_)	0.15	0.02	0.12	0.16	−0.11	0.16
Stability of female tenure (*τ*_*f*_)	0.13	0.03	0.11	−0.09	0.16	0.14
Pair-bond stability* (*ε*)	0.03	0.28	0.29	−0.03	−0.32	−0.04

### Pair-bond stability and the production of full siblings

All else being equal, pair-bond stability had little influence on average within-group relatedness; across simulations, there was almost no correlation between the pair-bond stability parameter (ε) and mean relatedness, *r*_*s*_ = 0.03. While it is certainly true that pair-bond stability increases the relatedness of the offspring born to the same mother, it is not the case that pair-bond stability will increase relatedness across a group containing multiple males and multiple females within which all paternity is within the group (as assumed in this model). Pair-bond stability does, however, influence the relative proportion of full and half-siblings, and across our simulations, pair-bond stability was positively correlated with the proportion of full siblings (*r*_*s*_ = 0.29) and negatively with the proportion of maternal half-siblings (*r*_*s*_ = −0.32). Pair bonding can have a particularly important effect on the distribution of sibling types under some conditions. For example, in a monotocous group containing multiple males and multiple females (*N*_*m*_ = 10, *N*_*f*_ = 10, *κ* = 1) with no reproductive skew in either sex (*α* = 0, *β *= 1), the proportion of full siblings produced over 4 reproductive cohorts (*n* = 4) under random mating (*ε* = 0.5) is 10 times lower than under monogamy (*ε* = 1) (0.75% of dyads at *ε* = 0.5 versus 7.5% of dyads at *ε* = 1; Fig. 1d).

## Discussion

Previous work has shown that groupmates will be more closely related when groups are smaller and when reproductive skew is greater (Altmann 1979; Lukas et al. 2005; Dyble and Clutton-Brock 2020, 2023). In this paper, I look beyond average within-group relatedness and instead consider the factors that determine the production of full siblings, maternal half-siblings, and paternal half-siblings within social groups. The model results suggest that the relative frequency of maternal and paternal half-siblings will be driven mostly by differences in sex ratio and reproductive skew. When there are relatively few males in the group, or when one male dominates mating opportunities, many offspring will share a father but not a mother and will, therefore, be paternal half-siblings. When there are relatively few females or when a single female monopolizes reproduction, most offspring will share a mother and be at least maternal half-siblings. The production of full siblings can occur through two routes: it can either be a consequence of very high levels of reproductive skew among both sexes (as seen in cooperatively breeding species) or a consequence of a high degree of pair-bond stability within groups containing multiple males and multiple females (as seen in humans). Other factors to influence the production of different kinship types are litter size (large litters increase the number of full siblings and decrease the proportion of half-siblings) and the number of juveniles cohorts (more juvenile cohorts results in more maternal half-siblings but fewer paternal half-siblings and full siblings).

As outlined above, the proportion of paternal versus maternal half-siblings is of interest because although the average relatedness to them is the same (typically *r* = 0.25), maternal kin are more readily identified (Langergraber et al. 2007; Widdig 2007; Perry et al. 2008). For full siblings, the major evolutionary relevance is that the production of full siblings (who are related to one another on average by the same degree as parents and offspring are, *r* = 0.5) is strongly associated with transitions to cooperative breeding (Hughes et al. 2008; Cornwallis et al. 2010; Lukas and Clutton-Brock 2012; Kramer and Russell 2014). While it is clearly the case that pair-bond stability increases relatedness among the offspring born to the same female, pair-bond stability does not increase the average degree of relatedness among juveniles born into a group containing multiple males and multiple females, as long as all paternity occurs with the group. This is because, for every full sibling dyad that is created, two half-sibling dyads are lost: at a group level, variance in relatedness increases with pair-bond stability, but average within-group relatedness does not.

The results relating to pair bonding have implications for understanding the evolution of human sociality. Humans are highly cooperative despite living in groups in which average genetic relatedness is low (Gintis et al. 2003; Hrdy 2009; van Schaik and Burkart 2010; Burkart et al. 2014; Lehmann et al. 2022). This is true not just for large urban societies where relatedness between individuals is effectively zero but also in small-scale societies; mean relatedness within hunter-gatherer and horticulturalist camps rarely exceeds *r* = 0.1 (Hill et al. 2011; Walker 2014; Dyble et al. 2021). This is a significantly lower level of relatedness than usually associated with highly cooperative social behavior (Lukas and Clutton-Brock 2018) and with the evolution of alloparental care in mammals (Briga et al. 2012). However, despite the low level of average relatedness in human groups, the relative proportion of full siblings in humans is very high and much higher than seen within chimpanzee communities: an initial literature search suggests that the proportion of siblings living in the same community that is full siblings (rather than half-siblings) ranges from 0% to 4% across 4 chimpanzee populations (Tai forest [Vigilant et al. 2001], Mahale [Inoue et al. 2008], Gombe [Walker et al. 2017], Ngogo [Langergraber et al. 2007]) but from 33% to 100% across 80 small-scale human communities (averaging ~60%) included in an analysis by Ellsworth and colleagues (2016). These values put the relative proportions of full siblings in human groups at levels more similar to those seen in cooperative breeders than in other plural breeding species. For example, for cooperative breeders, the proportion of siblings that are full siblings is 85% in African wild dogs (*Lycaon pictus*) (Girman et al. 1997), 85% in Ethiopian wolves (*Canis simensis*) (Randall et al. 2007), and 60% in meerkats (*Suricata suricatta*) (C. Duncan, personal communication). The proportions among plural breeders appear to be much lower: 19.6% in European badgers (*Meles meles*) (Dugdale et al. 2008), 12.1% in Colombian white-faced capuchin (*Cebus capucinus*) (Perry et al. 2012), and 4.8% in Olive baboons (*Papio anubis*) (Lynch et al. 2017), noting that some of these studies aggregate data at a group level and others at a population level. More systematic future analyses could explore these patterns across mammals more thoroughly.

It is important to note that the focus of the model presented here is the frequency of sibling dyads among the offspring born into a social group. These sibling relationships represent the highest degree of genetic relatedness that will be seen among members of the same generation (offspring of monozygotic multiple births excepted) and will go on to determine the degree of relatedness among non-dispersing adult groupmates. It is, therefore, concerned with the principal determinants of the kinship composition of groups. However, the present analysis could be extended to consider kinship between adults directly, exploring the consequences of factors such as longevity, overlapping generations, dispersal, and extra-group paternity on the kinship composition of the group and to consider the factors influencing the production of more distant kin such as cousins, aunts, and uncles.

To understand the evolution of social behavior, we need to understand variation in social organization and the kinship structure of groups (West et al. 2021). While average genetic relatedness within social groups is strongly associated with social behavior across mammals (Briga et al. 2012; Lukas and Clutton-Brock 2018), it is important to consider not only a single point estimate of average within-group relatedness but also the distribution of kinship within groups, as done here and in a recent empirical paper (Pereira et al. 2023). In addition, it is important to consider how males and females differ in their relatedness to groupmates and how an individuals’ relatedness to their group changes as they age. Consideration of these age and sex differences in relatedness (described as kinship dynamics) have been explored in recent work (Koster et al. 2019; Croft et al. 2021; Ellis et al. 2022; Chen et al. 2023; Du et al. 2023) and have been argued to contribute to divergent fitness interests that can have implications for social and life-history evolution (Johnstone and Cant 2008, 2010). Future theoretical models could potentially integrate kinship dynamics with models of the kinship composition of groups.

## Supplementary Material

arae032_suppl_Supplementary_Materials

## Data Availability

The model script is available in the electronic supplementary material.
