# Supplementary material for: Explaining variation in the kinship composition of mammal groups
Source: Behav Ecol. 2024 Apr 17;35(3):arae032. doi: 10.1093/beheco/arae032 (PMC11059295; doi:10.1093/beheco/arae032)
Supplement: arae032_suppl_Supplementary_Materials [file arae032_suppl_supplementary_materials.pdf]

Supplemental Material

Explaining variation in the kinship composition of mammal groups

Mark Dyble

md479@cam.ac.uk

Estimating relatedness among juveniles

As in Dyble and Clutton-Brock (2020), relatedness among juveniles, r_j, is estimated as $0.25(P+M)$ where here $P = P(full)+P(pat)$ and $M = P(full)+P(mat)$. Relatedness through the grandparental generation (r_j') is then added as follows:

$$r_j' = \frac{r_j}{2} + P\left(0.25 - \frac{r_j}{4}\right) + M\left(0.25 - \frac{r_j}{4}\right)$$

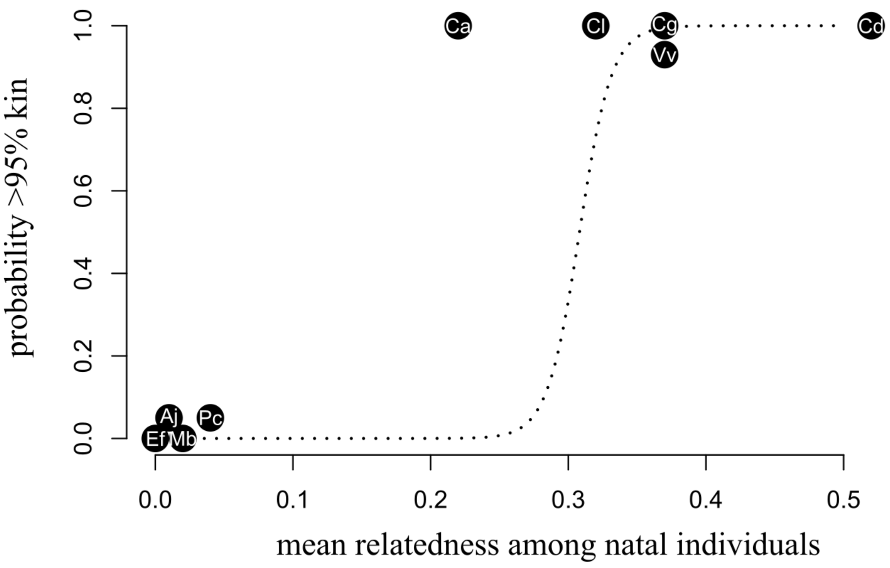

Figure S1: Exploring the relationship mean relatedness among natal individuals and the characterisation of a group as either ‘related’ or ‘mix-related’. Dotted line is the predicted relationship from a logistic regression model predicting the probability of >95% of juveniles being related (sharing at least one grandparent) and the mean relatedness among juveniles across the model results. Solid dots are empirical data points compare the classification of species from Pereira et al. (2023) as ‘related’ or ‘mix-related’ with estimates of relatedness among natal females for those species compiled by Briga et al. (2012). The empirical estimates are not used in the production of the logistic regression model. Nine of the 18 species listed in Pereira et al. (2023) are included in Briga et al (2012). These are: *Cryptomys damarensis* (Damaraland Mole-rat, *Cd*, mean r = 0.52, ‘related’), *Ctenodactylus gundi* (Common gundi, *Cg*, mean r = 0.37, ‘related’), *Papio cynocephalus* (Yellow baboon, *Pc*, mean r = 0.04, ‘mix-related’), *Myotis bechsteinii* (Bechstein's bat, *Mb*, mean r = 0.02, ‘mix-related’), *Eptesicus fuscus* (Big brown bat, *Ef*, mean r = -0.05, plotted as r = 0, ‘mix-related’), *Artibeus jamaicensis* (Jamaican fruit bat, *Aj*, mean r = 0.01, ‘related’), *Canis lupus* (Grey wolf, *Cl*, mean r = 0.32, ‘related’), *Cuon alpinus* (Dhole, *Ca*, mean = 0.22, ‘related’), *Vulpes vulpes* (Red fox, *Vv*, mean = 0.37, ‘related’). Closely plotted points are jittered.

Figure S2: Histograms showing the frequency with which the proportion of siblings in the model is predicted to exceed >50% full siblings or more than 1/3rd full siblings, maternal half siblings, or paternal siblings across variation in a set of parameters, where all other parameters are sampled randomly. If a parameter had no effect on sibling composition, a uniform distribution would be expected. Though not linear, all relationships are monotonic, justifying the use of a Spearman's correlation coefficient, as reported in the main text.

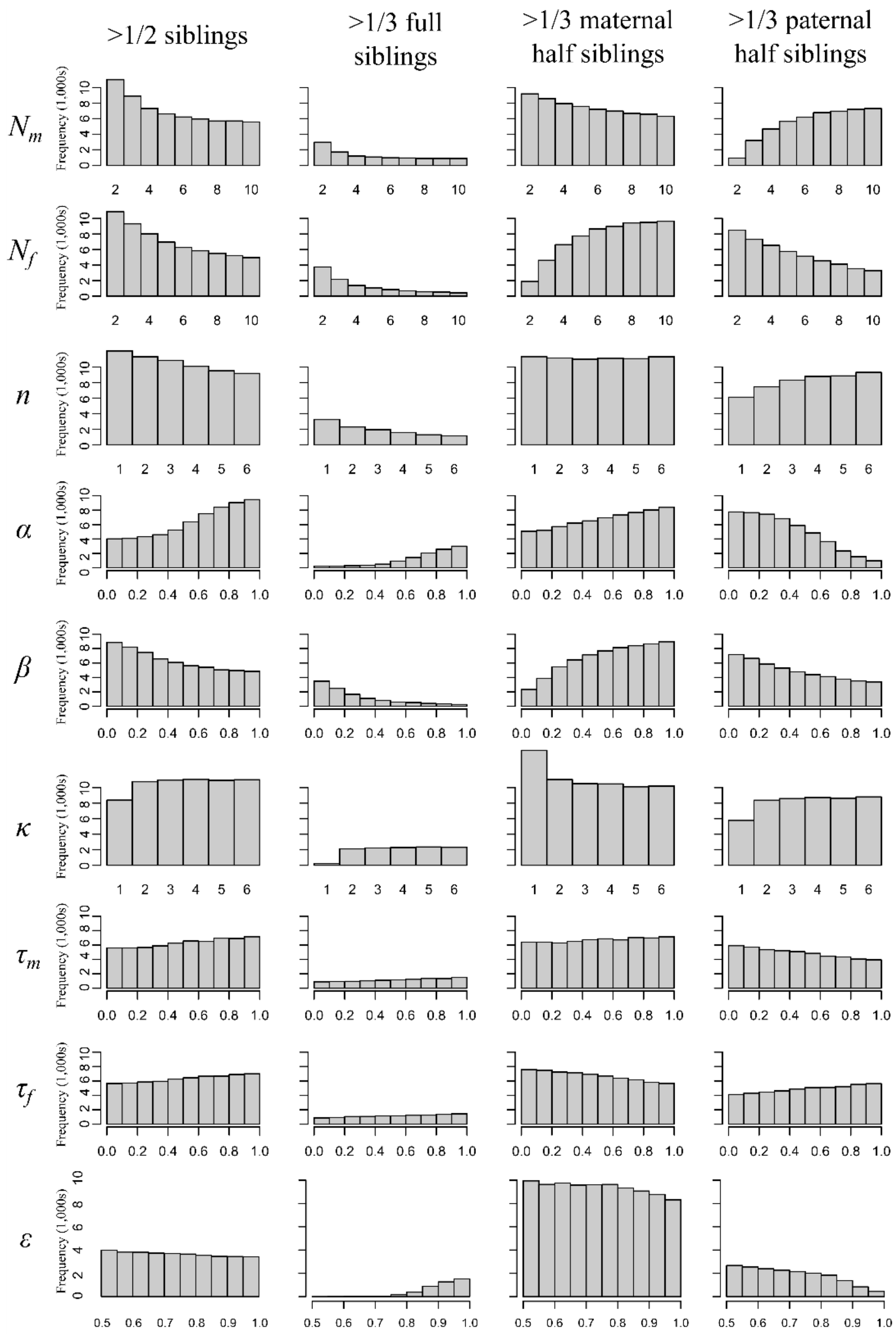

Table S1: Spearman's rank correlation coefficients (ρ) between parameters and measures of relatedness and kinship composition from the second set of simulations in which pair-bond stability is varied randomly across the range $0.5 \leq \varepsilon < 1$, and where the range of values for N_f and β is restricted to reduce the frequency of impossible combinations ($5 \leq N_f \leq 10$; $0.5 \leq \beta \leq 1$), with the few remaining impossible parameter combinations excluded (~2% of the total). Results for 10^5 simulations.

	Spearman's rank correlation coefficients with:					
Parameter:	mean relatedness	sd in relatedness	% full sibs	% paternal sibs	% maternal sibs	% sibs
Number of males (N_m)	-0.46	-0.27	-0.32	-0.47	0.28	-0.46
Number of adult females (N_f)	-0.19	-0.37	-0.36	0.08	-0.37	-0.13
Juvenile cohorts (n)	-0.16	0.00	-0.13	-0.18	0.39	-0.14
Male reproductive skew (α)	0.70	0.12	0.37	0.72	-0.34	0.73
Female subordinate reproduction (β)	-0.11	-0.22	-0.22	0.04	-0.17	-0.07
Litter size (κ)	0.10	0.30	0.32	-0.07	0.14	0.04
Stability of male tenure (τ_m)	0.19	0.17	0.30	0.15	-0.32	0.15
Stability of female tenure (τf)	0.05	0.08	0.08	-0.02	0.10	0.04
Pair-bond stability (ε)	0.03	0.28	0.29	-0.03	-0.32	-0.04

References cited

Briga M, Pen I, Wright J. 2012. Care for kin: within-group relatedness and allomaternal care are positively correlated and conserved throughout the mammalian phylogeny. Biol Lett. 8(4):533–536. doi:10.1098/rsbl.2012.0159.

Dyble M, Clutton-Brock TH. 2020. Contrasts in kinship structure in mammalian societies. Behavioral Ecology. 31(4):971–977.

Pereira AS, De Moor D, Casanova C, Brent LJN. 2023. Kinship composition in mammals. R Soc open sci. 10(7):230486. doi:10.1098/rsos.230486.
